# Impact of COVID-19 Pandemic and Associated Restrictions on Vitamin D Status in a Large Cohort of Italian Children and Adolescents

**DOI:** 10.3390/medicina60010065

**Published:** 2023-12-29

**Authors:** Roberto Antonucci, Nadia Vacca, Beatrice Biasia, Cristian Locci, Maria Pina Dore, Giovanni Mario Pes, Angela Bitti

**Affiliations:** 1 Pediatric Clinic, Department of Medicine, Surgery and Pharmacy, University of Sassari, 07100 Sassari, Italy; 2 Internal Medicine Section, Department of Medicine, Surgery and Pharmacy, University of Sassari, 07100 Sassari, Italygmpes@uniss.it (G.M.P.); 3 Laboratory of Azienda Ospedaliero-Universitaria, 07100 Sassari, Italy

**Keywords:** vitamin D, seasonal variation, sex-related difference, COVID-19 pandemic, children

## Abstract

*Background and Objectives*: Vitamin D is synthesized in the skin upon sunlight exposure, showing variations with season and latitude. We aimed to investigate the influence of age, sex, and season on vitamin D status in a large pediatric cohort during the COVID-19 pandemic period and the corresponding pre-pandemic period. *Materials and Methods*: Retrospective data concerning subjects aged < 18 years were extracted anonymously from the large database of a reference laboratory hospital (Sassari, Northern Sardinia, Italy). Serum 25-hydroxyvitamin D [25(OH)D] levels measured during the pre-pandemic period (1 March 2018 to 30 September 2019) were compared with those detected during the pandemic period (1 March 2020 to 30 September 2021). *Results:* A total of 2317 samples from subjects aged < 18 years were included in the analysis, 1303 (47.9% females) of which were collected in the pre-pandemic period and 1014 (51.3% females) in the pandemic period. No significant differences in 25(OH)D levels were found between the two periods, whereas, in children aged < 2 years, levels were higher than those in children aged 11–16 years (*p* < 0.05). Monthly levels of 25(OH)D between pre-pandemic and pandemic periods did not differ, although significant differences were detected across months (*p* < 0.0001). Similarly, 25(OH)D values did not differ significantly between males and females in both periods. Marked seasonal variations were observed in males and females across all age groups. *Conclusions:* Serum vitamin D levels and their season-related variations were not significantly affected by the COVID-19 pandemic and associated restrictions in a large cohort of Italian children and adolescents.

## 1. Introduction

Vitamin D is a pro-hormone that plays an essential role in the regulation of calcium and phosphorus metabolism, and is a crucial determinant of bone health during childhood and adolescence. Furthermore, vitamin D has important “extra-skeletal” effects, including regulation of cell proliferation and differentiation, immunomodulation, and regulation of several hormonal systems [1].

The role of vitamin D in bone metabolism and its ability to prevent rickets has been recognized since the beginning of the last century [2]. The function of vitamin D is especially essential in the developmental age when skeletal growth occurs; in this phase, hypovitaminosis D may negatively affect bone mineralization.

Regarded in the early years as a simple “vitamin”, in recent decades, several experimental studies have documented that the biologically active form of vitamin D [1,25(OH)2D] circulates in the blood and functions as an essential hormone, involved in the regulation of cell proliferation, immunomodulation, glucose metabolism, and human longevity [1,3]. In clinical practice, total body vitamin D status is usually evaluated by measuring serum 25-hydroxyvitamin D [25(OH)D] levels, given its long half-life, relatively abundant circulating concentrations, and resilience to fluctuations in parathyroid hormone (PTH) [4].

Sunlight ultraviolet-B (UV-B) radiation (wavelength 290–315 nm) is essential for promoting the conversion of cholesterol into vitamin D. This discovery dates back to nearly a century ago [5,6]. Since then, evidence has accumulated that insufficient exposure to sunlight is one of the most important causes of vitamin D deficiency [7].

The seasonal variations of solar radiation reaching the earth’s surface are reflected in the amount of electromagnetic energy that reaches the exposed skin of humans, thus resulting in the serum 25(OH)D level seasonal pattern, with a maximum peak in summer and a minimum in spring [8,9]. Although the seasonal variations of vitamin D levels have been known for a long time, in-depth studies investigating sex- and age-related variations are mainly limited to relatively small cohorts [10].

In December 2019, a new infectious disease caused by the SARS-CoV-2 virus appeared in Wuhan, China [11]. Within a few months, the virus spread worldwide, reaching a pandemic level and causing a health emergency [12]. Stringent public health measures were imposed in several countries to contain the infection.

In line with the WHO recommendations, the Italian government adopted restrictive measures to contain virus circulation. A lockdown period was imposed between March and May 2020, and citizens were prohibited from leaving their homes “except for movements motivated by proven work needs, state of necessity, or health reasons.” The measures adopted to limit the COVID-19 pandemic included: (i) replacement of face-to-face teaching in a classroom setting with virtual teaching, (ii) closure of many workplaces, and (iii) suspension of meetings and events leading to crowding [13]. Therefore, the prolonged home confinement of children and adolescents due to the lockdown measures deprived them of the opportunity to attend school and to physically engage with their peers [14]. This led to a substantial reduction in their outdoor recreation activities and sunlight exposure [15], thus increasing the risk of developing vitamin D deficiency or insufficiency [16].

Sardinia is an Italian island in the western Mediterranean Sea, located between 39° N and 41° N latitude, with a temperate climate. Sardinia has an average monthly Global Horizontal Irradiance of 4.04 kWh/m^2^/day and an average monthly Direct Normal Irradiance of 3.9 kWh/m^2^/day [17], with seasonal variations comparable to that of the Mediterranean countries located at the same latitude [18].

There is scarce data available regarding the influence of sex, age, and season on vitamin D status during the COVID-19 pandemic in children and adolescents.

The purpose of this study was to investigate the effect of age, sex, and season on serum vitamin D levels in a large cohort of Italian children and adolescents during the COVID-19 pandemic period and the corresponding pre-pandemic period.

## 2. Materials and Methods

This retrospective observational study was performed using routinely collected data extracted anonymously from an electronic database of the Reference Laboratory at the Azienda Ospedaliero-Universitaria of Sassari. Sassari is a medium-sized city located in the northwest of the island of Sardinia, Italy (latitude, 40°43′ N, longitude 8°33′ E). This laboratory is the reference for all Hospitals and inhabitants of Sassari city (nearly 120,000 inhabitants) and its hinterland (90,000 inhabitants).

Data on serum 25(OH)D concentrations from inpatient and outpatient subjects aged < 18 years referred to the laboratory in the periods 1 March 2018–30 September 2019 (pre-pandemic period) and 1 March 2020–30 September 2021 (pandemic period) were collected. The date of each measurement was also obtained, although, for each subject, only the earliest one was considered for the analysis. Demographic information included sex and age.

Monthly changes in mean serum 25(OH)D levels in the subjects studied were analyzed considering a 12-month time interval for both the pre-pandemic period (1 March 2018–28 February 2019) and the pandemic period (1 March 2020–28 February 2021).

In addition, a comparison was made between the mean 25(OH)D values of subjects tested during the lockdown period (1 March 2020–31 May 2020) and those of subjects tested during the same period of the previous year (1 March 2019–31 May 2019), divided by sex.

The serum levels of 25(OH)D were assessed by using the immune-chemiluminescence Liaison^®^ 25 OH Vitamin D Total Assay (CLIA, DiaSorin Spa, Saluggia (VC), Italy) following the manufacturer’s instructions [19].

Exclusion criteria: (i) 25(OH)D values higher than 100 ng/mL were excluded from the analysis as they were considered well above the reference range and attributed to analytical errors or to therapeutic overdose; (ii) when multiple tests were available for a single subject, those following the earliest one were excluded.

For the purpose of this study, data on serum vitamin D values were categorized in line with the classification published in 2016 by the “Global Consensus Recommendations on Prevention and Management of Nutritional Rickets” [20]. In particular, vitamin D status was classified as sufficiency (serum 25(OH)D > 20 ng/mL), insufficiency (serum 25(OH)D, 12–20 ng/mL), and deficiency (serum 25(OH)D < 12 ng/mL).

### Statistical Analysis

Quantitative variables are presented as means and standard deviations. Qualitative or non-parametric data were summarized as absolute and relative (percentage) frequencies. Measurements of serum 25(OH)D levels corresponding to the tested samples from 1 March 2018 to 30 September 2021 were stratified by sex, age, and season. Differences between serum 25(OH)D values were analyzed with a two-tailed Student’s *t*-test for unpaired data or one-way analysis of variance where appropriate. All statistical computations were performed using SPSS statistical software (version 22.0, Chicago, IL, USA). *p* values < 0.05 were considered statistically significant.

## 3. Results

Measurements of serum 25(OH)D concentration were available from 2601 eligible individuals aged < 18 years. Following the exclusions described above, serum 25(OH)D level measurements in 2317 subjects were included in the analyses. The study population was stratified into two groups based on the measurement date: a first group included subjects tested for serum vitamin D from 1 March 2018 to 30 September 2019 (pre-pandemic period), and a second group included subjects tested from 1 March 2020 to 30 September 2021 (pandemic period). The “pre-pandemic” group consisted of 1303 subjects, of whom 624 (47.9%) were females, while the “pandemic” group included 1014 subjects, of whom 520 (51.3%) were females. The proportions of subjects with serum vitamin D deficiency (<12 ng/mL), insufficiency (12–20 ng/mL), and sufficiency (>20 ng/mL) in both groups are shown in Table 1.

No significant differences in vitamin D status were detected between the subjects tested in the two periods considered.

The serum vitamin D levels in the “pre-pandemic” and “pandemic” groups, stratified by age, are shown in Table 2. The only significant differences in serum 25(OH)D levels between the two groups were found in children aged one year, six years, and 12 years, respectively. In particular, statistically higher 25(OH)D levels were detected in 1-year-old children (*p*-value: 0.007) and in 6-year-old children (*p*-value: 0.049) included in the “pandemic” group. In comparison, higher 25(OH)D levels were found in 12-year-old children (*p*-value: 0.023) included in the “pre-pandemic” group.

The mean serum vitamin D level was within the sufficient range in the pre-pandemic period (24.46 ng/mL) and the pandemic period (24.24 ng/mL). In both groups, the one-way analysis of variance with age as a factor revealed significant differences (*p*-values: 0.02 in the pre-pandemic group and <0.0001 in the pandemic group), and the multiple comparison test revealed differences between subjects younger than 2 years on the one hand (higher serum vitamin D levels), and subjects aged 11-16 years on the other (lower serum vitamin D levels).

Serum 25(OH)D levels in subjects tested in the pre-pandemic and pandemic periods, divided by sex and age, are indicated in Table 3 and Table 4, respectively.

No significant difference was found in the mean values of vitamin D between the subjects tested in the two study periods, divided by sex. However, both in the “pre-pandemic” and “pandemic” groups, ANOVA showed significant differences across age groups (*p*-value = 0.006), and, in particular, significantly higher values of vitamin D (*p*-value = 0.002) were observed in “pre-pandemic” males aged 16 years (Table 3), while, in the “pandemic” group, serum vitamin D levels were found to be significantly higher (*p*-value = 0.045) in females aged 3 years (Table 4).

**Figure 1:**
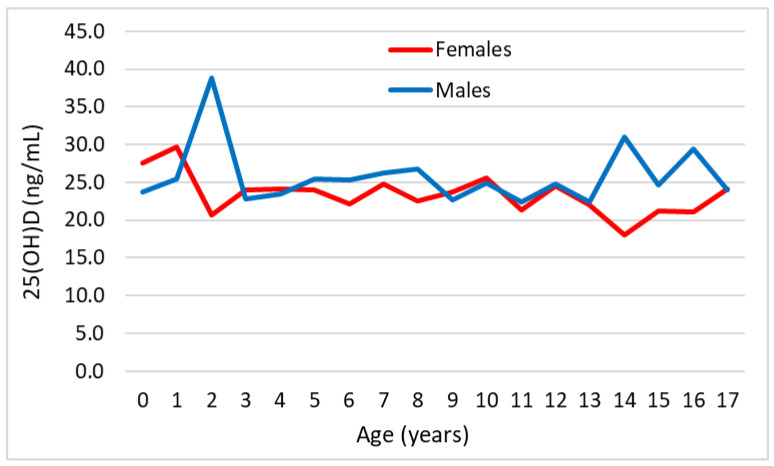
Graphical representation of mean serum 25(OH)D values in subjects included in the “pre-pandemic” group as a function of age and stratified by sex.

**Figure 2:**
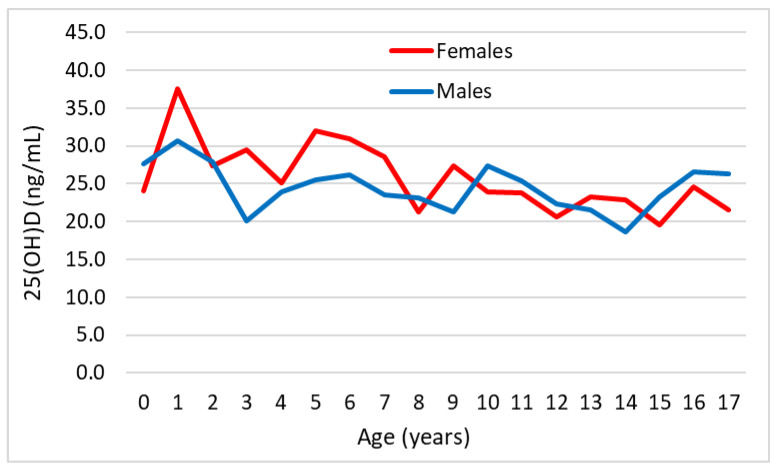
Graphical representation of mean serum 25(OH)D values in subjects included in the “pandemic” group as a function of age and stratified by sex.

The mean serum vitamin D values in subjects included in the “pre-pandemic” and “pandemic” groups, as a function of age and stratified by sex, are shown in Figure 1 and Figure 2, respectively.

Figure 2 shows that, during the “pandemic”period, serum 25(OH)D levels were consistently lower, albeit non-statistically significant, in males than females, particularly in subjects younger than 10 years.

**Figure 3:**
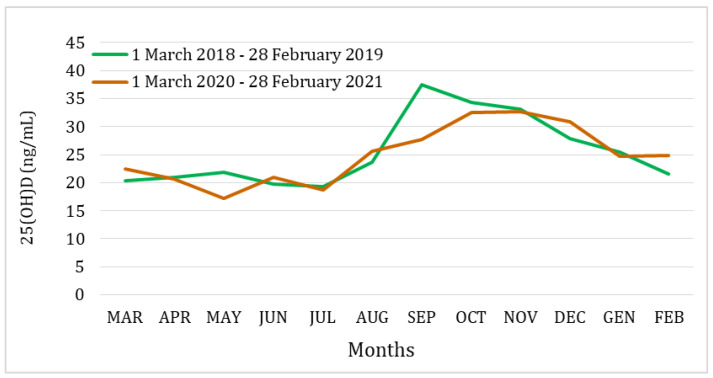
Monthly changes in mean serum 25(OH)D values of subjects tested over a 12-month period before the pandemic (1 March 2018–28 February 2019, green line) and during the pandemic (1 March 2020–28 February 2021, brown line).

The monthly changes in mean serum 25(OH)D levels of subjects tested over a 12-month period spanning from 1 March 2018 to 28 February 2019, before the pandemic, and from 1 March 2020 to 28 February 2021, during the pandemic, are shown in Figure 3. No significant monthly differences were found between the subjects tested in the two study periods, with the exception of May and September when vitamin D levels were found to be significantly lower in the “pandemic” group (*p*-value = 0.025).

The one-way analysis of variance, with the month of the year as a factor, showed a significant global difference (*p*-value < 0.0001) in mean serum 25(OH)D levels across all months, in both periods considered.

**Figure 4:**
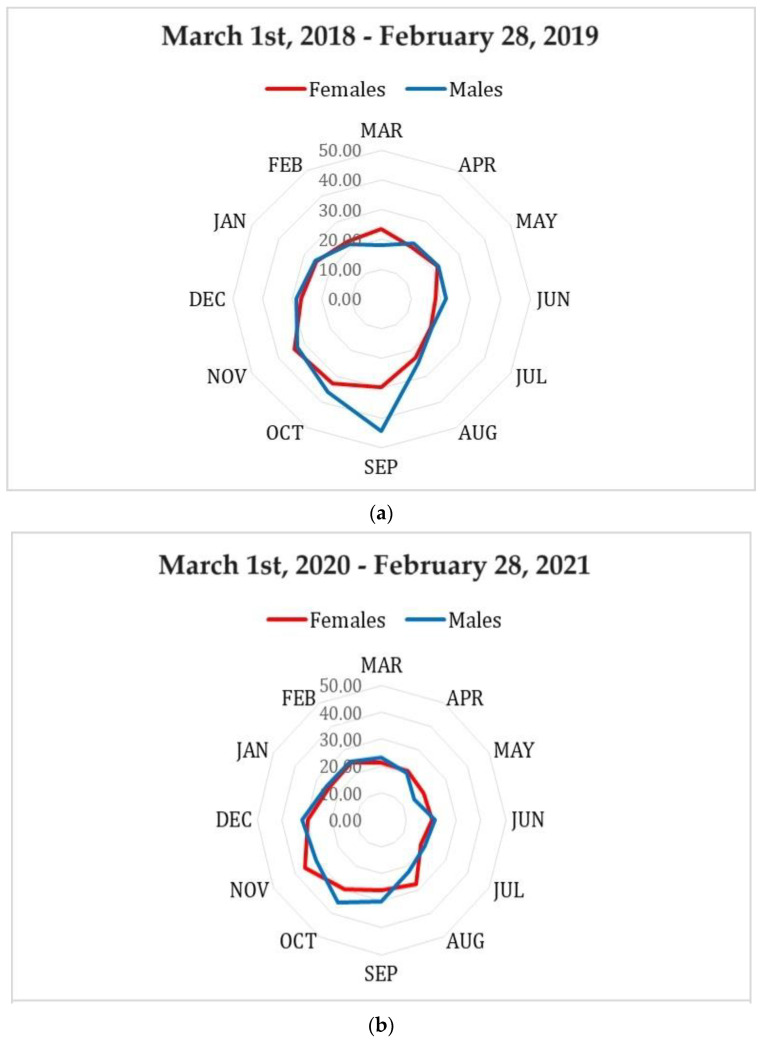
Monthly changes in mean serum 25(OH)D values (ng/mL) of subjects tested over a 12-month period before the pandemic (1 March 2018–28 February 2019), divided by sex (**a**); Monthly changes in mean serum 25(OH)D values (ng/mL) of subjects tested over a 12-month period during the pandemic (1 March 2020–28 February 2021), divided by sex (**b**).

The monthly changes in mean serum 25(OH)D levels of subjects tested over a 12-month period before the pandemic (1 March 2018–28 February 2019) and during the pandemic (1 March 2020–28 February 2021), divided by sex, are illustrated in Figure 4a and b, respectively.

The difference in monthly 25(OH)D values between males and females was not significant for both study periods, although a borderline significant difference (*p*-value = 0.055) was observed among subjects tested in the pre-pandemic period.

The comparison between the mean values of 25(OH)D among subjects tested in the lockdown period (1 March 2020–31 May 2020) and those detected among subjects tested in the same period of the previous year (1 March 2019–31 May 2019), divided by sex, did not reveal any statistically significant difference (Table 5).

## 4. Discussion

In humans, vitamin D status depends on several factors, the most important of which is sunlight exposure [6]. Solar irradiance varies throughout the year, from a maximum in the summer months to a minimum in the winter months; these variations are mirrored by comparable fluctuations in the serum concentration of vitamin D. Seasonal variations in vitamin D levels have been described in numerous populations [21,22,23,24,25,26]; however, sex- and age-related variations have been reported only in a few observational studies [27,28].

In our study, all data were retrieved from a large database available in the laboratory of a medium-sized hospital on the Italian island of Sardinia, to investigate the impact of age, sex, and season on serum vitamin D levels in a pediatric cohort, during the COVID-19 pandemic period and a corresponding pre-pandemic period. On this island, the amount of UV-B radiation absorbed by the skin all year round is potentially able to allow the majority of subjects exposed to sunlight to produce adequate amounts of vitamin D (cholecalciferol).

In line with these considerations, our study highlighted an annual variation in mean serum 25(OH)D concentration parallel to the variation in solar irradiance, with a time lag of about 2–3 months.

In Italy, since the beginning of the COVID-19 pandemic, the government and health authorities adopted strict measures to contain the spread of SARS-CoV-2. In particular, home confinement measures may have increased the risk of developing vitamin D deficiency, particularly in children and adolescents.

In the present study, the impact of these restrictive measures on serum vitamin D levels was assessed in a large pediatric population living in northern Sardinia. When comparing the pre-pandemic period (1 March 2018–30 September 2019) to the pandemic period (1 March 2020–30 September 2021), no statistically significant difference was found in the proportions of subjects with deficient, insufficient, and sufficient serum vitamin D levels. The mean serum vitamin D values in children recruited during the pre-pandemic period were in the sufficiency range (24.46 ng/mL); the mean vitamin D values measured during the pandemic period were also in the sufficiency range, and very similar to those observed in the pre-pandemic period (24.24 ng/mL). Moreover, in both “pre-pandemic” and “pandemic” groups, vitamin D levels were found to be significantly higher in subjects younger than 2 years as compared to older children.

Hypovitaminosis D has been frequently found in children even in the pre-COVID-19 era, as documented by several epidemiological studies [29]. The National Health and Nutrition Examination Survey (NHANES) studies, conducted in the USA, revealed a high proportion of vitamin D deficiency and insufficiency in the pediatric age [30,31]. In Italy, several studies have shown a high prevalence of vitamin D deficiency, especially among immigrant and adopted infants and children [32,33,34,35]. However, other studies carried out in the Tuscany and Veneto regions reported mean serum vitamin D levels in the sufficiency range. For example, Vierucci et al. [33], in Pisa (43° N), found mean 25(OH)D levels of 20.7 ng/mL, while Franchi et al. [35], in Verona (45° N), reported mean levels of 21 ng/mL. Reasonably, the higher levels of vitamin D seen in specific Italian geographical areas such as northern Sardinia may depend on multiple factors including latitude, lifestyle, diet, and dressing style among others. In fact, Sardinia and Tuscany are located at about 40° N latitude, and thus are exposed to higher UV-B irradiation as compared to other Italian regions, implying a greater capacity for vitamin D skin synthesis.

Several studies reported that the percentage of children with vitamin D insufficiency (i.e., 25(OH)D below 20 ng/mL) progressively increases from childhood to adolescence [33,36,37,38]. In line with this finding, our results revealed higher vitamin D values in the age group < 2 years as compared to the older age groups; this was found in both pre-pandemic and pandemic periods, thus ruling out the impact of COVID-19 pandemic on age-related variations in serum vitamin D concentrations.

In the literature, no sex-related differences in serum vitamin D levels have been reported in the pediatric age [33,39,40,41]. In the study by Vierucci et al. [33], adolescent age, female sex, and urban residence were not associated with an increased risk of hypovitaminosis D. More recently, Mosca et al. [38], in a large retrospective study, found no statistically significant differences in age (*p*-value = 0.83) and sex (*p*-value = 0.94) distribution between children hospitalized in the “pre-COVID” period and those hospitalized in the “post-COVID” period. Our results are consistent with these data, as serum vitamin D levels were similar in males and females, in both the pre-pandemic and pandemic periods.

Few literature studies have investigated the impact of the restrictions imposed during the COVID-19 pandemic on serum 25(OH)D levels in the pediatric population. In 2020, the study by Yu et al. [16] revealed that the proportion of children with vitamin D deficiency (serum 25(OH)D levels < 50 nmol/L) during the pandemic period was higher than that observed in previous years. In Poland, Rustecka et al. [42] also found that the percentage of children with serum 25(OH)D levels < 20 ng/mL was higher in the pandemic period than in the pre-pandemic period (17% vs. 14%). From our data analysis, mean vitamin D concentrations were not significantly lower in subjects tested during the pandemic period considered. Moreover, a subanalysis was performed to precisely evaluate the impact of lockdown and associated restrictions on the vitamin D status in our study population. For this purpose, we compared serum 25(OH)D levels in subjects tested during the lockdown period (1 March 2020–31 May 2020) to those in subjects tested in the corresponding period of 2019 (1 March 2019–31 May 2019). Again, no significant difference was found between the two groups.

In 2020, Yu et al. [16] highlighted an increasing trend of vitamin D deficiency with age. More recently, Wong et al. [43] have found pre-pandemic 25(OH)D levels to be significantly higher than pandemic ones. This difference was more significant in older children (7–24 months) than in younger children (2–6 months). In our study, the serum levels of 25(OH)D measured in the pandemic period showed a reduction with increasing age, albeit remaining in the sufficiency range.

Rustecka et al. [42] pointed out that the characteristic seasonal pattern of serum vitamin D levels, resulting from the different sun exposure throughout the year, disappeared during the COVID-19 pandemic. Mosca et al. [38] found significantly lower (*p*-value = 0.02) serum vitamin D levels in the summer during the post-pandemic period compared to the pre-pandemic period, with a smoothing of the vitamin D values peak in the summer season. Conversely, the data from our study show that the typical seasonal variations in serum vitamin D levels were maintained even during the COVID-19 pandemic. In fact, in both pre-pandemic and pandemic periods, the lowest vitamin D levels were found during the winter and spring. In contrast, vitamin D levels significantly increased during the summer and autumn. In just two months (i.e., May and September), 25(OH)D levels were lower in the pandemic than in the pre-pandemic period.

Several limitations of this study need to be mentioned. First, it was a retrospective study, which precludes the possibility of causal inferences. Second, only the pediatric population of a single geographical area was investigated, somewhat limiting the generalizability of the results. Third, given that the analysis was performed using data from an electronic database, the characteristics of the patients for whom the test was requested were unknown; as a consequence, some variables that impact vitamin D status, such as body mass index, lifestyle, diet, and physical activity, were unfortunately not available. On the other hand, we think that the confounding effect of a possible vitamin D supplementation has been attenuated mainly by the exclusion from the analysis of all samples following the earliest one obtained from a single individual.

## 5. Conclusions

In conclusion, the present study shows that serum vitamin D levels in a large cohort of Italian children and adolescents living in Northern Sardinia were not significantly affected by the restrictive measures imposed during the pandemic period under study. In fact, vitamin D concentrations largely remained in the sufficiency range during this period and did not differ significantly from those observed in the pre-pandemic period. We can hypothesize that the latitude at which the children and adolescents investigated live, as well as their lifestyle, with the possibility of spending long time periods outdoors, could be protective factors against the development of hypovitaminosis D. Findings from this study shed light for the first time, on vitamin D status in a large cohort of Italian children and adolescents living in northern Sardinia, with a special focus on the changes in vitamin D levels associated with the COVID-19 pandemic and concomitant restrictions. Further studies with larger cohorts of children and adolescents from different geographical areas are required to assess the real impact of the COVID-19 pandemic and associated control measures on vitamin D status.

## Figures and Tables

**Table 1:** Vitamin D status in subjects aged < 18 years tested in the pre-pandemic and pandemic periods.

25(OH)D Range	Pre-Pandemic Group (*n* = 1303)		Pandemic Group (*n* = 1014)		*p*-Value *
	*n*	%	*n*	%	
Deficiency (<12 ng/mL)	140	10.7	131	12.9	0.106
Insufficiency (12–20 ng/mL)	383	29.4	299	29.5	0.960
Sufficiency (>20 ng/mL)	780	59.9	584	57.6	0.255

* Chi-square test.

**Table 2:** Serum 25(OH)D levels in subjects aged < 18 tested in the pre-pandemic and pandemic periods, respectively, and stratified by age.

Age (Years)	Pre-Pandemic Group (*n* = 1303)		Pandemic Group (*n* = 1014)		*p*-Value *
		25(OH)D (ng/mL)		25(OH)D (ng/mL)	
	*n*	Mean ± S.D.	*n*	Mean ± S.D.	
0	107	25.58 ± 12.15	119	25.90 ± 13.70	0.854
1	79	27.16 ± 10.19	35	33.43 ± 13.06	0.007
2	53	30.58 ± 53.15	44	27.70 ± 12.63	0.726
3	79	23.06 ± 9.84	37	23.19 ± 13.37	0.955
4	79	23.72 ± 11.09	31	24.45 ± 7.63	0.737
5	91	24.84 ± 10.52	34	26.71 ± 9.85	0.370
6	68	23.93 ± 8.42	34	27.85 ± 11.08	0.049
7	63	25.57 ± 13.14	35	25.69 ± 11.07	0.965
8	56	24.86 ± 9.23	32	22.31 ± 9.22	0.217
9	78	23.10 ± 8.22	42	24.74 ± 16.31	0.466
10	75	25.28 ± 10.02	62	25.55 ± 9.65	0.874
11	64	21.84 ± 8.07	45	24.73 ± 14.50	0.186
12	64	24.66 ± 8.69	82	21.39 ± 8.36	0.023
13	69	22.20 ± 9.77	67	22.40 ± 8.88	0.901
14	69	24.97 ± 46.45	78	20.92 ± 10.02	0.454
15	81	22.52 ± 9.72	84	20.54 ± 11.03	0.223
16	73	23.30 ± 10.26	65	25.35 ± 13.03	0.303
17	55	24.04 ± 9.49	88	23.00 ± 11.60	0.579
Total	1303	24.46 ± 17.90	1014	24.24 ± 11.84	0.735

* Student’s *t*-test.

**Table 3:** Serum 25(OH)D levels in subjects aged < 18 years tested in the pre-pandemic period, divided by sex and age.

Age (Years)	Females		Males		*p*-Value
	25(OH)D (ng/mL)		25(OH)D (ng/mL)		
	*n*	Mean ± S.D.	*n*	Mean ± S.D.	
0	53	27.51 ± 13.43	54	23.69 ± 10.53	0.104
1	32	29.72 ± 9.80	47	25.43 ± 10.19	0.066
2	24	20.71 ± 11.30	29	38.76 ± 70.64	0.222
3	21	23.95 ± 11.83	58	22.74 ± 9.11	0.632
4	34	24.09 ± 11.77	45	23.44 ± 10.68	0.800
5	37	24.00 ± 9.12	54	25.41 ± 11.43	0.534
6	29	22.10 ± 5.84	39	25.28 ± 9.76	0.124
7	29	24.79 ± 13.63	34	26.24 ± 12.87	0.668
8	25	22.52 ± 7.35	31	26.74 ± 10.24	0.089
9	33	23.70 ± 8.79	45	22.67 ± 7.85	0.588
10	42	25.52 ± 8.00	33	24.97 ± 12.26	0.814
11	33	21.36 ± 7.79	31	22.35 ± 8.45	0.627
12	38	24.58 ± 8.97	26	24.77 ± 8.44	0.932
13	31	22.00 ± 10.67	38	22.37 ± 9.11	0.877
14	32	18.03 ± 6.47	37	30.97 ± 62.93	0.251
15	51	21.24 ± 9.26	30	24.70 ± 10.24	0.122
16	54	21.13 ± 9.06	19	29.47 ± 11.18	0.002
17	26	24.08 ± 8.71	29	24.00 ± 10.30	0.976
Total	624	23.46 ± 10.04	679	25.37 ± 22.82	0.055

**Table 4:** Serum 25(OH)D levels in subjects aged < 18 years tested in the pandemic period, divided by sex and age.

Age (Years)	Females		Males		*p*-Value
	25(OH)D (ng/mL)		25(OH)D (ng/mL)		
	*n*	Mean ± S.D.	*n*	Mean ± S.D.	
0	59	24.12 ± 13.01	60	27.65 ± 14.24	0.161
1	14	37.50 ± 14.49	21	30.71 ± 11.58	0.134
2	17	27.41 ± 11.71	27	27.89 ± 13.39	0.905
3	12	29.50 ± 18.55	25	20.16 ± 9.00	0.045
4	14	25.07 ± 7.68	17	23.94 ± 7.78	0.689
5	6	32.00 ± 11.35	28	25.57 ± 9.33	0.149
6	12	31.00 ± 14.91	22	26.14 ± 8.24	0.227
7	15	28.60 ± 12.45	20	23.50 ± 9.66	0.181
8	14	21.29 ± 7.97	18	23.11 ± 10.24	0.587
9	24	27.38 ± 18.63	18	21.22 ± 12.21	0.231
10	32	23.87 ± 7.93	30	27.33 ± 11.06	0.160
11	19	23.84 ± 14.80	26	25.38 ± 14.54	0.729
12	44	20.57 ± 9.63	38	22.34 ± 6.60	0.341
13	34	23.26 ± 9.66	33	21.52 ± 8.04	0.424
14	42	22.88 ± 9.72	36	18.64 ± 10.01	0.062
15	61	19.51 ± 8.96	23	23.26 ± 15.14	0.166
16	39	24.54 ± 13.22	26	26.58 ± 12.89	0.541
17	62	21.60 ± 9.19	26	26.35 ± 15.68	0.080
Total	520	23.90 ± 11.92	494	24.59 ± 11.76	0.352

**Table 5:** Serum 25(OH)D values in subjects tested from 1 March 2019 to 31 May 2019 (group A) and in those tested during the Italian lockdown period (1 March 2020 to 31 May 2020, group B), stratified by sex.

Sex	Group A (*n* = 202)			Group B (*n* = 78)			*p*-Value *
	*n*	25(OH)D (ng/mL)		*n*	25(OH)D (ng/mL)		
		Mean	S.D.		Mean	S.D.	
Females	97	19.10	7.10	41	18.93	10.57	0.909
Males	105	23.35	38.18	37	18.43	11.18	0.442
Total	202	21.31	27.98	78	18.69	10.80	0.094

* Student’s *t*-test.

## Data Availability

The datasets generated and/or analyzed during the current study are available from the corresponding author upon reasonable request.
